# A modified technique of single-incision laparoscopic hepaticojejunostomy for children with choledochal cysts

**DOI:** 10.1186/s12893-019-0499-3

**Published:** 2019-04-11

**Authors:** Di Xu, Kunbin Tang, Shaohua He

**Affiliations:** Pediatrics Surgery Department, Fujian Provincial Hospital, Fuzhou, 350001 China

**Keywords:** Choledochal cyst, Laparoscopy, Anastomosis, Minimally invasive surgery, Transumbilical

## Abstract

**Background:**

To present a modified laparoscopic surgical technique that works to optimize the surgical view in laparoscopic total excision of choledochal cyst in pediatric patients.

**Methods:**

From June 2015 to June 2017, a total of 48 pediatric cases of choledochal cyst were admitted. Their age ranged from 15 month to 8 years (average 3.5 years). The Todani types were: type I (*n* = 32) and type IVa (*n* = 16), according to the diagnostic criteria of ultrasound, abdominal computed tomography (CT) and magnetic resonance cholangiopancreatography (MRCP).

**Results:**

Total cystic excision with hepaticoenterostomy was accomplished laparoscopically in 48 cases with our transumbilical single -incision method without conversion to open surgery. Average duration of operation was 200 min (range 170–240 min), average intraoperative blood loss was 9 ml (range 6–14 ml) without the need for blood transfusion. The 72-h postoperative ultrasound reported no abdominal effusion, when the intraperitoneal drainage tube was removed. There was no postoperative complication during the 6 months of follow-up.

**Conclusions:**

We accomplished the same postoperative outcome in laparoscopic total cyst excision with our modified method as that with conventional laparoscopic surgery. This technique allows the operator to have a stabilized surgical view without needing to rely on an assistant to hold up the liver lobe for larger operative space.

## Background

Since Farello et al. [1] first reported laparoscopic surgery for choledochal cyst (CC) excision in 1995, minimally invasive surgery has been increasingly used in the surgical intervention of CCs, which are congenital disorders manifested as cystic dilatation of the biliary ducts. CCs are primarily diagnosed before the age of 10 [2]. Pediatric CC is rare in western countries but common among East Asian populations, and affects girls much more often than boys.

Conventionally, the standard approach in open surgery for CC treatment includes total cyst excision and Roux-en-Y hepaticoenterostomy, preferably hepaticojejunostomy [3]. In China, laparoscopic total cyst excision with Roux-en-Y hepaticojejunostomy was first reported in the treatment of congenital CC in 2002, since which it has increasingly become the method of choice in the management of pediatric CC in some large hospitals [4]. The Roux-Y jejunojejunostomy anastomosis is typically performed extracorporally with the jejunum taken out through an umbilical incision. In recent years, single-incision laparoscopic cyst excision and Roux-en-Y hepaticojejunostomy (SILH) has been widely adopted for better cosmetic results and quicker recovery, and postulated to be comparable with conventional laparoscopic methods in surgical outcome [5, 6].

However, laparoscopic surgery for cyst excision is a technically challenging procedure and largely depends on the experience of the surgeon. Since 2015, we have adapted to laparoscopic CC excision in our pediatric department using a modified transumbilical single-incision method, and achieved satisfactory postoperative outcome. We present in this paper a technique used in our single-incision laparoscopic surgery for total CC excision that allows the operator to have a stable and enlarged surgical view without relying on a surgical assistant to expose the operative target or to provide a steady view.

## Methods

### Clinical data

A total of 48 pediatric patients of congenital CC underwent total cyst excision via transumbilical SILH in our department during June 2015 and June 2017. Among these, 35 cases were female and 13 male, 15 months to 8 years of age (average 3.5 years). Preoperative ultrasound, computed tomography (CT), and magnetic resonance cholangiopancreatography (MRCP) were performed to confirm the diagnosis of CC. According to the Todani classification [7], 32 cases were type I and 16 type IVa.

### Surgical steps

Routine preoperative intestinal lavage was performed, indwelling gastric tube and catheter to empty the stomach, colon and bladder.

The patient was placed in a supine position with head lifted at 30° and slightly tilted to the left, upper abdomen and lumbar elevated, intravenous-inhalation general anesthesia administered with caudal block.

**Fig. 1:**
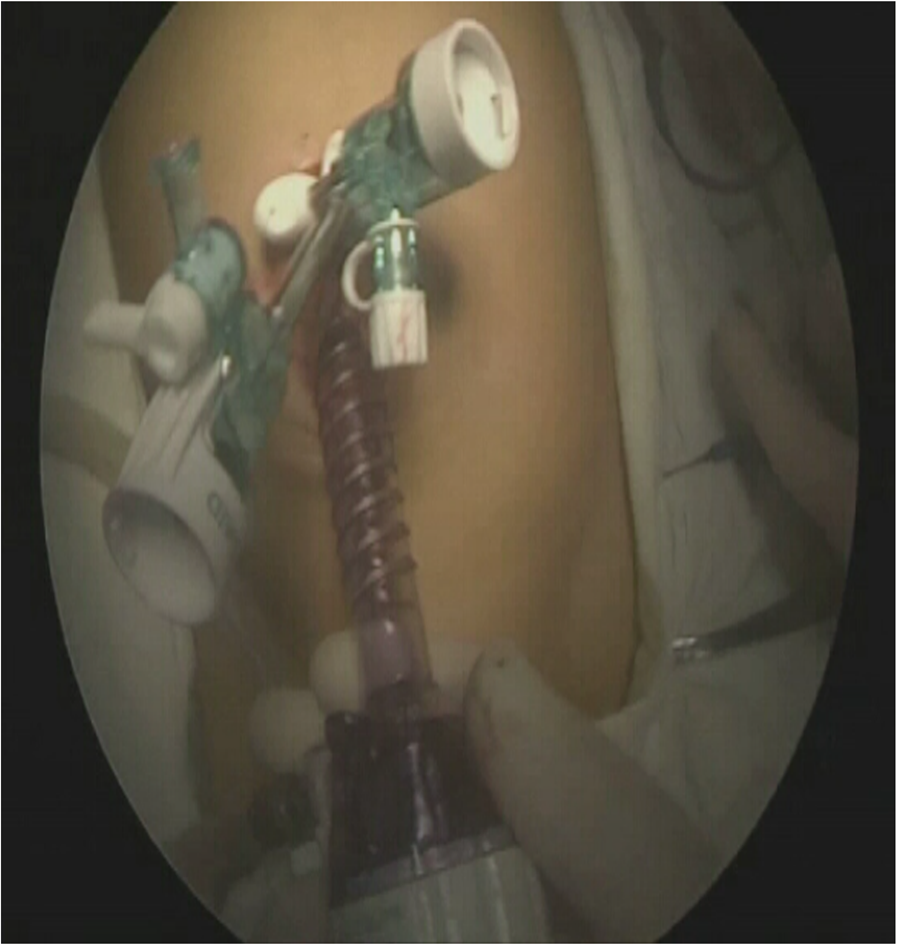
Through a 3-cm incision at the umbilicus, a 10-mm trocar, a 5-mm trocar, and a 3-mm trocar were placed

A 3 cm incision was made at the umbilicus, a 10 mm trocar placed through the navel wheel, a 5 mm trocar and a 3 mm trocar was placed 2 cm above the umbilicus on each side, 45° outward (Fig. 1).

Carbon dioxide pnumoperitoneum was established at a pressure of 10-12 mmHg.

**Fig. 2:**
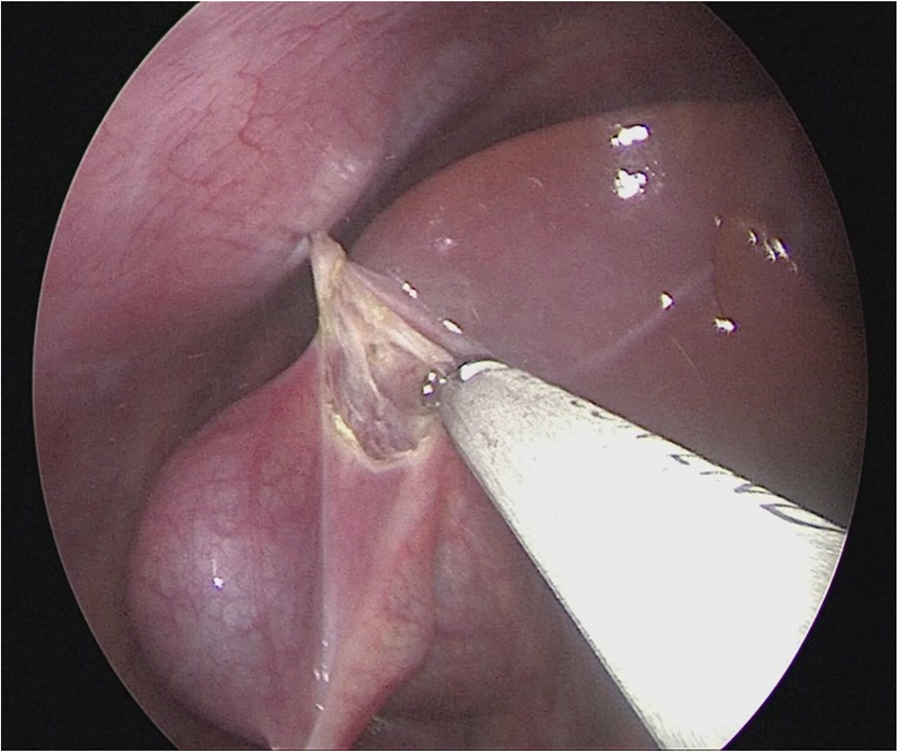
The liver was lifted to the abdominal wall and stabilized so that the operative field was enlarged to facilitate visualization of the hepatic porta

Insert 2–0 PDS sutures through below the xiphoid, to the right of the falciform ligament of liver (through the right hypochondrium), suture and lift the round ligament, then pull out the sutures to the left of the falciform ligament of liver; in the same manners, insert and pull out another round of sutures to lift the tissue of the gallbladder fundus; hence the liver was pulled to the abdominal wall and stabilized at two ends, and the operative field enlarged, facilitating optimal visualization of the porta hepatis (Fig. 2).

**Fig. 3:**
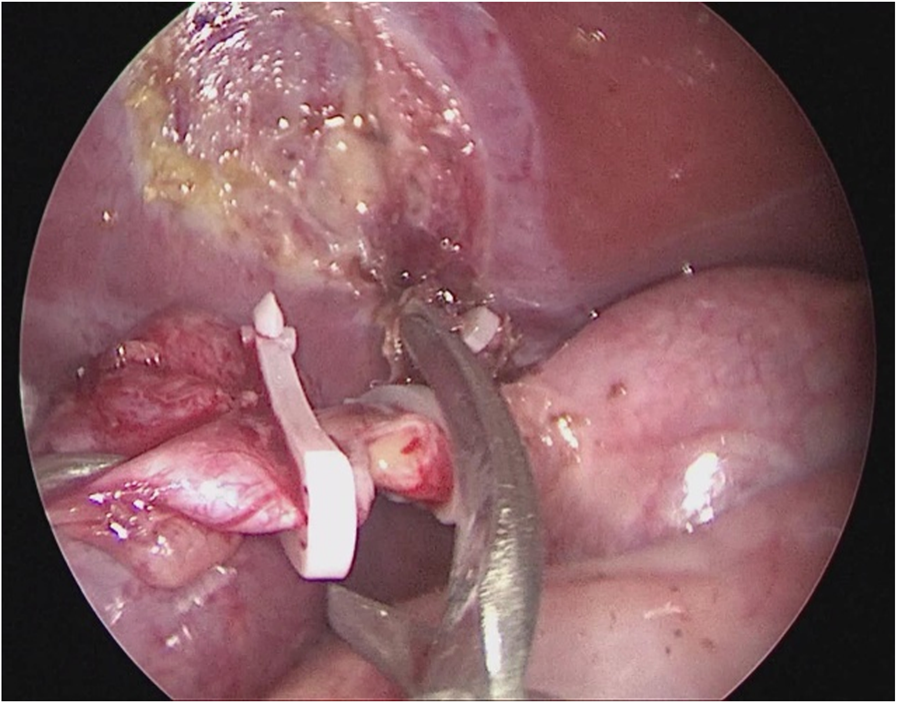
The cyst was separated, and cystic arteries ligated with bulldog clamps

**Fig. 4:**
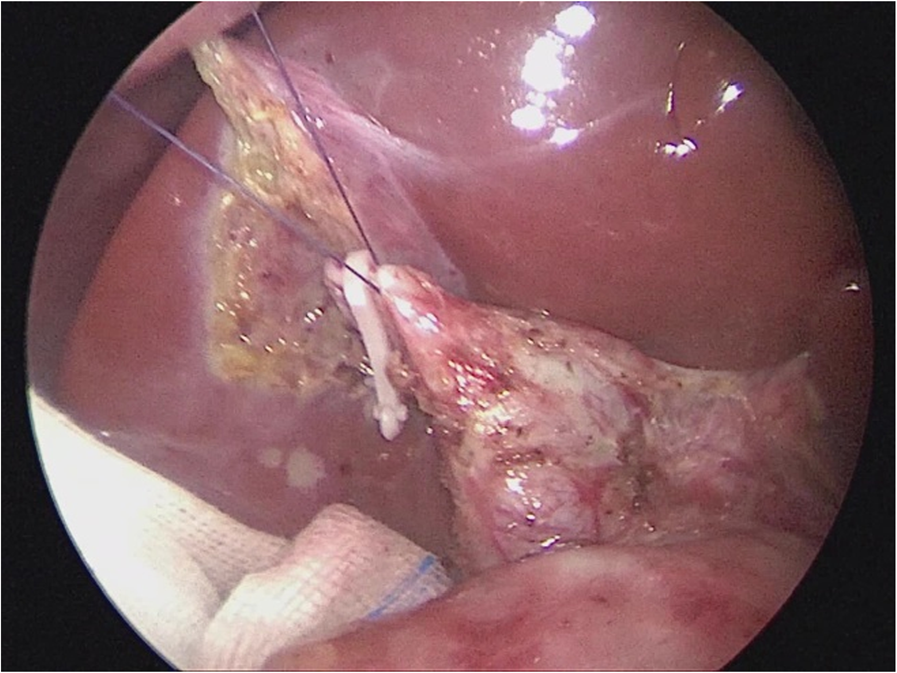
The cyst was separated from the peritoneum and exposed, which was made easy with the liver lifting and resulted traction

**Fig. 5:**
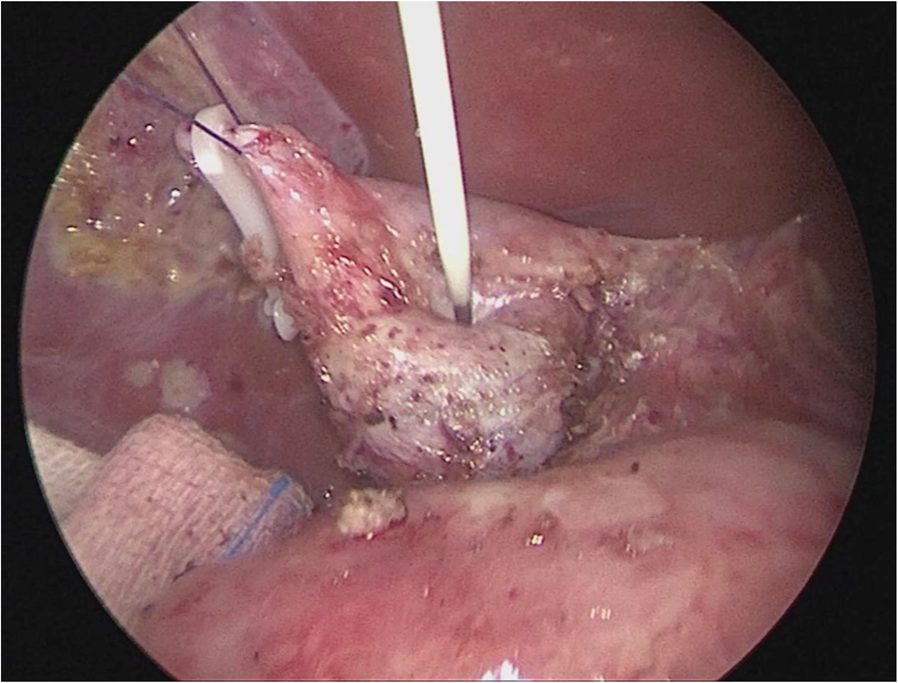
The cyst wall was punctured and bile drained

Loosen the cyst from the gallbladder and from the duodenum with an ultrasonic scalpel, detach, ligate the cystic arteries with clamps (Fig. 3), then disassociate the gallbladder to the junction of the cystic duct and the common bile duct, detach the peritoneum fromthe anterior CC wall to expose thecyst, then puncture the cyst and drain the bile (Figs. 4 & 5); in similar manners, separate the posterior cyst wall with the ultrasonic scalpel tightly kept to the cyst wall (be careful not to damage the portal vein), transect the cyst.

Pull the duodenum with forceps, the ultrasonic scalpel kept to the cyst wall, and separate the CC wall towards the thinning distal end of the cyst converging with the pancreatic duct, clip the distal end of the common bile duct with a hemo-lock, then resect the distal cyst wall. In a similar fashion, separate the proximal part of the cyst wall with an ultrasonic scalpel, to its normal junction with the hepatic duct; identify the opening of the left and right hepatic duct, then transversely dissect and resect the proximal cyst.

**Fig. 6:**
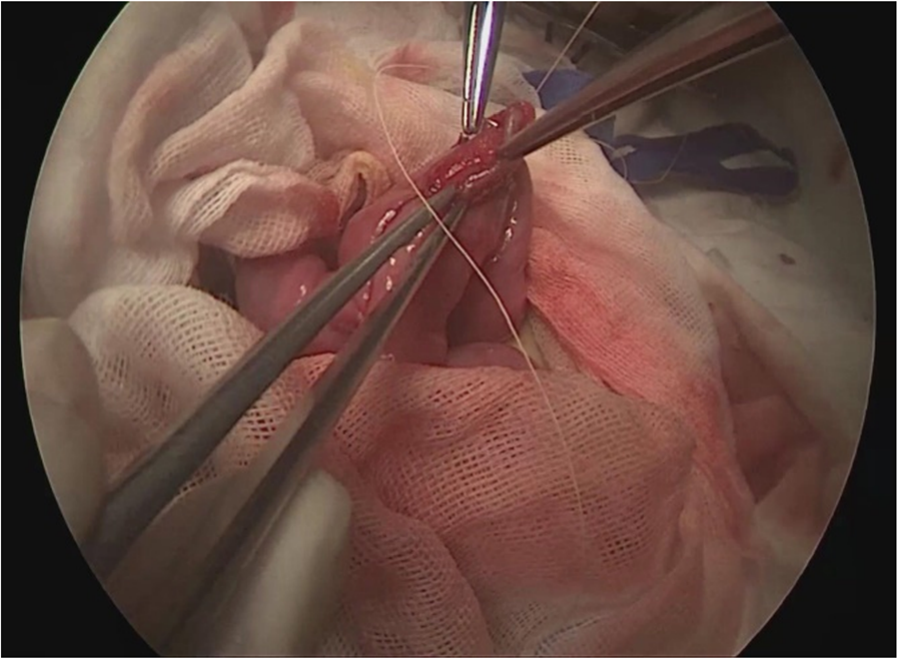
The side-to-side hepaticojejunostomy was completed with extracorporeal anastomosis

Flip the transverse colon up, identify the Treitz ligament, 25 cm from which to be the planned incision site; cut open the umbilical area and take out the jejunum, transecting it at the planned site with a linear stapler; fold the proximal jejunum, side-to-side with the distal jejunum 20 cm from the transection point, overlapping about 4 cm, fixing the two ends each with a stitch; on the overlapped sides, make a 1 cm opening each along the lateral wall at the anti-mesenteric border, place the cartridge seat and anvil, one in each opening, completing the anastomosis of the overlapping jejunum; then suture the opening site of the anti-mesenteric border with 5–0 Vicryl sutures to complete the side-to-side hepaticojejunostomy anastomosis (Fig. 6).

**Fig. 7:**
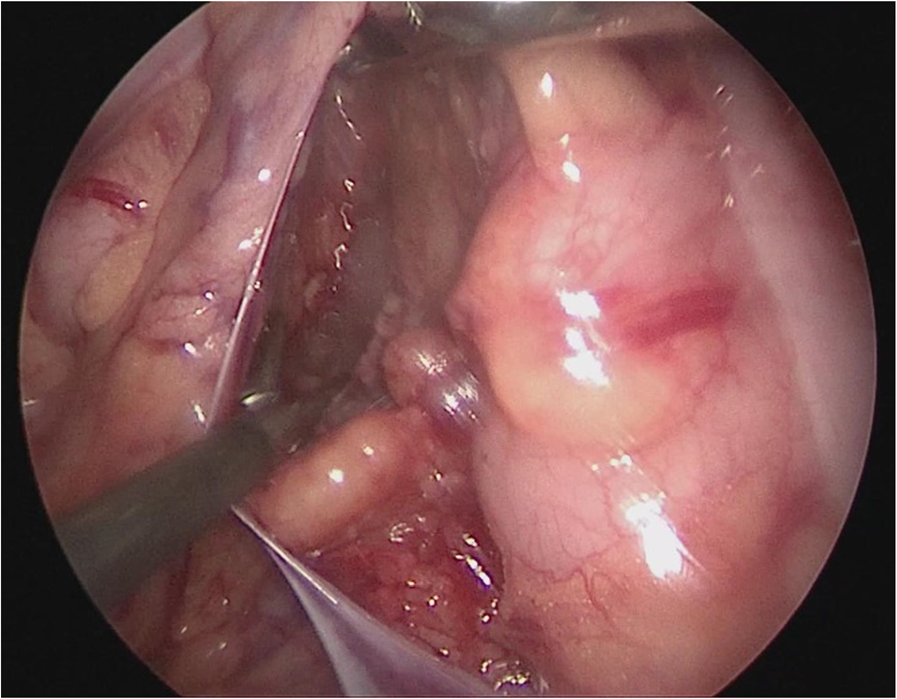
The hepatic Roux limb was placed and secured to the hilum via the tunnel behind the colon

Place the jejunum back in order in the peritoneal cavity; open up the vessel-free area to the right of the colon artery, place a suitable length of the Roux limb via the tunnel behind the colon, then to the hilum, secure its position (Fig. 7).

**Fig. 8:**
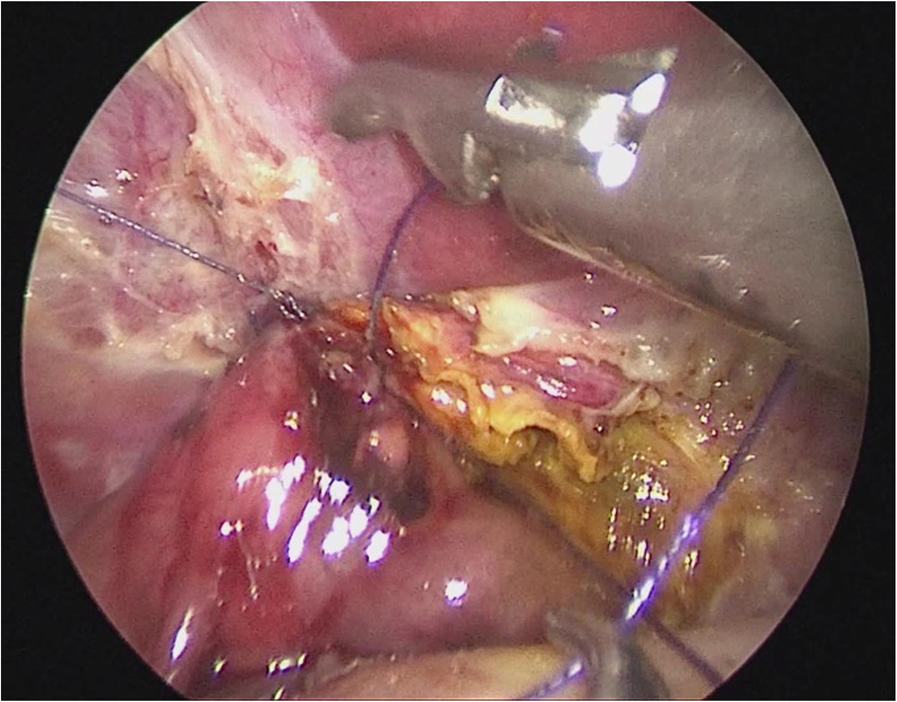
The anastomosis of the common hepatic duct with the jejunum

Cut open the jejunal wall at the anti-mesenteric edge, insert 4–0 PDS sutures through the right hypochondrium and tape the jejunum to the right side of the common hepatic duct, then retract the sutures through the abdominal wall to pull up the common hepatic duct. Adjust the lifting and traction to facilitate anastomosis; then use continuous stitches to tape the posterior wall of the common hepatic duct to the back wall of the jejunum with 4–0 absorbable barbed sutures; in the same manners, perform anastomosis of the anterior wall of the common hepatic duct and the front wall of the jejunum (Fig. 8).

Rinse the peritoneal cavity, then guide the drainage tube through the Trocar hole on the right of the umbilicus to the Winslow hole.

**Fig. 9:**
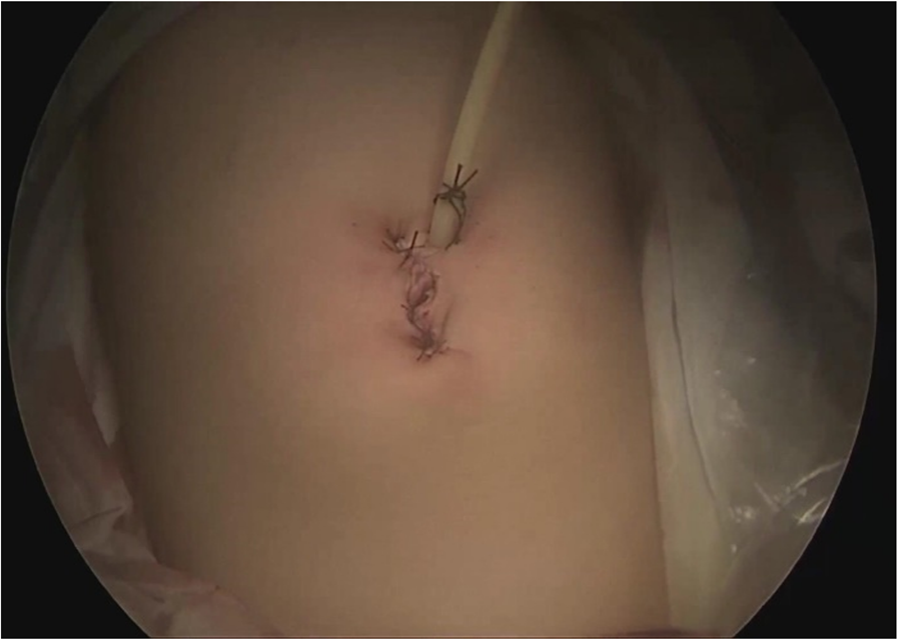
Postoperative umbilical incision

Confirm there is no hemorrhage, release pressure, remove trocar, suture the incision. Postoperative umbilical incision was 2.5-3 cm in length (Fig. 9).

## Results

All patients underwent laparoscopic complete excision of congenital CC, with Roux-en-Y jejunojejunostomy and hepaticojejunostomy. Operative time was 170–240 min (average 200 min); intraoperative blood loss was 6-14 ml (average 9 ml). No intraoperative or postoperative transfusion was needed; and there was no conversion to open surgery. The hepaticojejunostomy anastomosis was 0.9–1.2 cm in diameter.

Abdominal drainage tube was removed three days after the procedure. No seroperitoneum was observed on postoperative ultrasound B examination. None of the patients had intraoperative complications. The pediatric patients resumed ambulation in 24 h, resumed food intake in 48 h, and resumed normal activity, pain from the incision completely relieved, in five days post-surgery. Length of hospital stay was 6–9 days, no infection of the wound was observed.

During the six months of follow-up, ultrasound scan found no dilatation of the common hepatic duct or the intrahepatic bile duct. No case of bile reflux gastritis or cholangitis was found.

## Discussions

The incidence of CC is much higher in East Asian than in western countries. Especially in Japan, reportedly 1:1000 live births is diagnosed with CC, and the female to male ratio is as high as 4:1 [8].

For congenital CC patients, the key in successful management is total cyst excision with correction of the pancreaticobiliary maljunction (PBM), and reconstruction of the biliary tract [3, 9]. Majority of the cases are, by the Todani classification, type I (cystic or shuttle dilatation of the common bile duct), and type IVa (fusiform dilatation with extensions of dilatations of the intrahepatic bile ducts) CCs, for which it is crucial to perform wide hilar hepaticojejunostomy following complete cyst excision [2, 10]. Farello was first to report in 1995 the use of EndoGIA and Endostapler in laparoscopic complete cyst excision in CC surgery, and the use of 4–0 interrupted suture and cellulose glue in bilioenteric anastomosis: patients recovered uneventfully and were discharged in seven days [1]. Diao et al. first reported transumbilical single-port laparoscopic surgery with Roux-en-Y hepaticojejunostomy in children with congenital CCs and had satisfactory outcome [5]. Improved cosmesis is only an added benefit with a single-port laparoendoscopic approach compared with multi-port surgery. With less invasion, patients can recover and resume to normal activity more quickly, experience less pain and shorter hospital stay, given that the surgery is performed with equivalent precision.

In recent years, increasingly more adoptions of the single-site laparoscopic approach have been reported for surgical management of CC, and other general surgical procedures, including cholecystectomy and transperitoneal pyeloplasty [11–13]. Yet, reports of the application of SILH in the pediatric population are limited, perhaps because the technique of single-port laparoscopic reconstruction performed on children requires experienced hands that can maneuver in the very limited operative field. Moreover, there is a lack of surgical equipment specifically designed for pediatric laparoscopic operations.

We have adopted a modified transumbilical single-incision laparoscopic technique that offers the surgeon more control of the operative field. The operator can have a better/enlarged and stabilized operative view compared with conventional laparoscopic methods in which a surgical assistant is needed to perform the duties of exposing the hilum and other intended surgical areas. With this technique, lifting sutures are applied to pull and hold the liver lobe and the surgical view is enlarged and stabilized, which is particularly useful in laparoendoscopic pediatric surgery where the surgical space is often covered and concealed.

In addition, this technique can help enlarge and stabilize the operative field by minimizing the movement of local tissue, which facilitates the handling of dissection and anastomosis. This technique is simple and easily reproducible. It can be accomplished by: a) stitch and lift the round ligament and the gallbladder fundus with suture before separating and dissecting the cyst; b) stitch and lift the common hepatic duct before the hepaticojejunostomy anastomosis. With the tension and traction created by the lifting, creases are minimized and anastomosis is easier to perform as there is less of a chance to miss a stitch.

However, the lifting suture is meant to hold stable the surgical view and to offer the operator more control of the surgical field. This technical modification does not change the nature or the essence of a SILH approach, but to offer a better surgical experience. We described the clinical outcome during the 6-month follow-up of our pediatric patients undergoing this version of SILH when it started to be performed as standard of care in our hospital in 2015. Long term outcome will need to be examined when the cohort is followed for an intended 10-year time frame.

Critical voices have also been heard, regarding the single-site approach in the management of CC [14]. More long term observations and studies would be needed to resolve the debate and it is beyond the scope of our current work on the technical profile.

## Conclusions

In summary, single-incision laparoscopic cyst excision with hepaticojejunostomy has become the mainstream surgical management for CC in China, where the condition is commonly presented in children. Our modified SILH method with a lifting technique offers a simple and reproducible tool for surgeons to have more control over the operative field and improved surgical experiences.

## Abbreviations

CC: Choledochal cyst
CT: Computed tomography
MRCP: Magnetic resonance cholangiopancreatography
PBM: Pancreaticobiliary maljunction
SILH: Single-incision laparoscopic cyst excision and Roux-en-Y hepaticojejunostomy
